# Acceleration and deceleration of quantum dynamics based on inter-trajectory travel with fast-forward scaling theory

**DOI:** 10.1038/s41598-022-14973-6

**Published:** 2022-06-24

**Authors:** Shumpei Masuda, Jacob Koenig, Gary A. Steele

**Affiliations:** 1 Research Center for Emerging Computing Technologies (RCECT), National Institute of Advanced Industrial Science and Technology (AIST), 1-1-1, Umezono, Tsukuba, Ibaraki 305-8568 Japan; 2 Kavli Institute of Nanoscience, Delft University of Technology, Lorentzweg 1, 2628 Delft, CJ The Netherlands

**Keywords:** Quantum physics, Quantum mechanics, Qubits, Theoretical physics

## Abstract

Quantum information processing requires fast manipulations of quantum systems in order to overcome dissipative effects. We propose a method to accelerate quantum dynamics and obtain a target state in a shorter time relative to unmodified dynamics, and apply the theory to a system consisting of two linearly coupled qubits. We extend the technique to accelerate quantum adiabatic evolution in order to rapidly generate a desired target state, thereby realizing a shortcut to adiabaticity. Further, we address experimental limitations to the rate of change of control parameters for quantum devices which often limit one’s ability to generate a desired target state with high fidelity. We show that an initial state following decelerated dynamics can reach a target state while varying control parameters more slowly, enabling more experimentally feasible driving schemes.

## Introduction

An essential ingredient to the further development of quantum technologies is the ability to rapidly and accurately control quantum systems in order to overcome the effects of decoherence. However, modification of the speed of quantum dynamics is often complex in general due to both the lack of a simple scaling property in the dynamics as well as the infinitely large parameter spaces which one must generally navigate^1^. Thus, both experimentally feasible and nontrivial scaling properties in quantum dynamics are highly desirable to simplify the controls which regulate the time evolution of quantum systems.

Fast-forward scaling theory (FFST) provides a systematic way for optimally designing control parameters which accelerate, decelerate, or reverse the dynamics of a quantum system^1,2^. The formalism of FFST has previously been extended with great effect to many-body^3^ and discrete systems^4–6^, systems of charged particles^7,8^, tunneling dynamics^9,10^, Dirac dynamics^11,12^ and for the acceleration of adiabatic dynamics^13–15^. The application of FFST to adiabatic dynamics can produce what are known as shortcuts to adiabaticity (STA) or assisted adiabatic transformations^2,13,14,16^. Protocols utilizing FFST with quantum, classical, and stochastic dynamics have also been previously proposed^17,18^.

When utilizing FFST, one can often obtain viable trajectories through the system’s state space which realize the user’s desired end state. However, as we will show in this paper, FFST is not applicable in some parameter regimes due to the lack of a viable speed-controlled trajectory. Therefore, modification of the theory is required to resolve this issue. In this paper, we introduce a novel method which we call inter-trajectory travel (ITT), to resolve such deficiencies. Thus, our work addresses a fundamental challenge in quantum dynamics: the ability to control the rate of change of a quantum state. To demonstrate the effectiveness of ITT, we apply the framework to accelerate and decelerate the time evolution of two-level systems. Furthermore, we use ITT to realize shortcuts to adiabaticity by generating approximately the same state as that which is achieved by slower, adiabatic dynamics.

We focus in particular on deceleration in this study in contrast to previous works which have been largely concerned with fast and extremely precise controls. Control parameters for fast and accurate state preparation often have rapidly varying time dependencies when designed by other protocols^19,20^. However, there are typically experimental limitations to the rate of change of control parameters of a given system under examination^21^. Naively scaling down the rate of change of control parameters will in general not produce the desired target state, leading to a loss of fidelity. Our method for deceleration can be used to find slower control parameters which reliably generate approximately the same target state in a longer time without iterative integration of the Schrödinger equation.

In order to introduce our method we consider the acceleration and deceleration of a linearly coupled two-qubit system as an example. Although any arbitrary qubit state can be generated through a sequence of distinct single and two-qubit gates, it is often more convenient if one can generate a desired target state with fewer control parameters. In our method, the same single control parameter is modified with respect to the reference dynamics. Thus, our method does not require sophisticated manipulation of several control parameters, such as X, Y, and Z rotations of the qubits, but rather control over only the resonance frequency of a single qubit.

## System

In order to illustrate our method we consider a system of two coupled qubits as a concrete example, for which the Hamiltonian is represented as

1 $$\begin{aligned} H/\hbar= & {} \omega _1(t) \sigma _1^\dagger \sigma _1 + \omega _2 \sigma _2^\dagger \sigma _2 + g\bigg (\sigma _1^\dagger \sigma _2 + \sigma _2^\dagger \sigma _1\bigg ), \end{aligned}$$

where $\sigma _i$, $\omega _{i}$ and *g* are the annihilation operator, angular frequency of qubit *i* and the coupling strength between the qubits, respectively. $\sigma _i$ can be represented as $\sigma _i=|0\rangle _i \langle 1|_i$ with the ground and excited states of qubit *i* denoted by $|0\rangle _i$ and $|1\rangle _i$, respectively. $\sigma _i$ satisfies $\{\sigma _i,\sigma _i^\dagger \}=\sigma _i\sigma _i^\dagger + \sigma _i^\dagger \sigma _i=1$.

**Figure 1:**
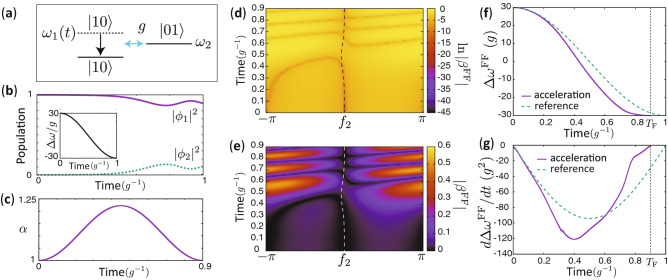
Schematic of the system, and speed-controlled and virtual trajectories for acceleration. (**a**) Schematic of the system. $\omega _1$ is decreased gradually, while $\omega _2$ and *g* are fixed. There is population transfer from $|10\rangle$ to $|01\rangle$. (**b**) Time dependence of population of $|m\rangle$ in the reference dynamics. The inset shows the time dependence of $\Delta \omega =\omega _1-\omega _2$. (**c**) Time dependence of the magnification factor $\alpha$ for the case of acceleration. The used parameters are $\Delta \omega _{0}=30g$, $T=g^{-1}$ and $T_{\text{F}}=0.9g^{-1}$. (**d,e**) $\ln |\beta ^{\text{FF}}|$ and $|\beta ^{\text{FF}}|$ as functions of $f_2$ and *t* for $T_{\text{F}}=0.9g^{-1}$. The dashed curves show a virtual trajectory. (**f**) Time dependence of $\Delta \omega ^{\text{FF}}=\omega _1^{\text{FF}}-\omega _2^{\text{FF}}$ for the virtual trajectory and $\Delta \omega$ for the reference dynamics. (**g**) Time dependence of $d\Delta \omega ^{\text{FF}}/dt$ and $d\Delta \omega /dt$.

Our system can be realized with a variety of platforms which enable frequency tunability of qubits. In particular, the field of circuit quantum electrodynamics^22–26^ in which a superconducting qubit’s transition frequency can be engineered to be modified by a magnetic flux threading its SQUID loop^27^, is a suitable candidate. A realization of the system is discussed in Supplementary Sect. S1.

We assume $\omega _1(t), \omega _2 \gg g$ for all times *t*. We require that $\omega _1(0)-\omega _2 \gg g$ and that the initial state of the system is the energy eigenstate which is approximately represented by $|10\rangle$, where the first and the second indices are for qubit 1 and qubit 2, respectively. Then, $\omega _1(t)$ is decreased gradually, while $\omega _2$ and *g* are fixed as illustrated in Fig. 1a. As the qubit frequencies near resonance, there is finite population transfer from $|10\rangle$ to $|01\rangle$ due to the coupling.

In the following analysis, we assume that the total time evolution of the system occurs on a timescale far shorter than the relevant coherence times of the qubits. Then, the dynamics of the system is confined to a subspace spanned by two states, $|{1}\rangle =|10\rangle$ and $|{2}\rangle =|01\rangle$. The state of the system under investigation is represented by

2 $$\begin{aligned} |\Psi (t)\rangle = \phi _{1}(t)|1\rangle + \phi _{2}(t)|2\rangle , \end{aligned}$$

with the Schrödinger equation written as

3 $$\begin{aligned} i\frac{d}{dt}\phi _{m}(t) = g \phi _{l}(t) + \omega _m(t) \phi _{m}(t), \end{aligned}$$

where hereafter $m,l\in \{1,2\}$ and $l\ne m$. The coherence time of superconducting qubits has been being improved. Nowadays, superconducting qubits with the coherence time of more than 10 $\upmu$s are routinely realized. For example, a relaxation time of $T_1=44$ $\upmu$s and dephasing time of $T_2=15$ $\upmu$s were reported in 2013^28^, while recently coherence times have reached hundreds of microseconds^29,30^. A typical reported value of the coupling strength between qubits is $g/2\pi =30$ MHz^31^. The longest control duration used in this study, $30g^{-1}=0.16$ $\upmu$s, is much shorter than average achievable coherence times. Therefore, we assume that the effect of decoherence is negligible in this study.

The dynamics of this system can be emulated also by a single qubit system under a drive after moving to a rotating frame and applying the rotating wave approximation (RWA) as explained in Supplementary Sect. S2.

## Fast-forward scaling theory

We derive the time dependence of $\omega _m$ which modifies the dynamics of the system, following the manner used in Ref. ^4^. The reference dynamics which is to be accelerated or decelerated is defined by $\phi _m(t)$ which satisfies Eq. (3). The target state is defined as $\phi _m(T)$ for $T>0$. We aim to generate the target state at a desired time $T_{\text{F}}\ne T$ from a given initial state which is the same as in the reference dynamics.

We write the wave function of the speed-controlled dynamics in terms of the wave function of the reference dynamics, $\phi _m(t)$, as

4 $$\begin{aligned} \phi _m^{\text{FF}}(t) = \phi _m(\Lambda (t)) e^{if_m(t)}, \end{aligned}$$

where $f_m(t)$ is an additional time-dependent phase and $\Lambda (t)$ is the scaled time defined by

5 $$\begin{aligned} \Lambda (t) = \int _0^t \alpha (t')dt'. \end{aligned}$$

In Eq. (4), $\phi _m(\Lambda (t))$ is the wave function of the ideal dynamics naively scaled with respect to time. Here, $\alpha$ is called the *magnification factor*. When $\alpha >1$, the time evolution of $\phi _m(\Lambda (t))$ is accelerated, while when $0<\alpha <1$ the dynamics are slowed and when $\alpha <0$, the dynamics are reversed. For instance, in the case where $\alpha =2$ the accelerated dynamics are twice as fast as the reference dynamics. However $\phi _m(\Lambda (t))$ cannot be realized when *g* is fixed. We introduce the additional phase $f_m(t)$ so that the state with the wave function in Eq. (4) can be realized even with fixed *g* (see Supplementary Sect. S3). The time dependence of $\alpha$ is chosen so that it satisfies

6 $$\begin{aligned} \Lambda (T_{\text{F}}) = T. \end{aligned}$$

Note that the wave function satisfies $\phi _m^{\text{FF}}(0) = \phi _m(0)$ and $\phi _m^{\text{FF}}(T_{\text{F}}) = \phi _m(T)$ if the additional phase vanishes at the initial and final time, $T_{\text{F}}$.

We assume that $\phi _m^{\text{FF}}$ is a solution of the Schrödinger equation:

7 $$\begin{aligned} i\frac{d}{dt}\phi _m^{\text{FF}}(t) = g \phi _{l}^{\text{FF}}(t) + \omega _m^{\text{FF}}(t) \phi _m^{\text{FF}}(t). \end{aligned}$$

The coupling strength is the same as in Eq. (3). We substitute Eq. (4) into Eq. (7), divide by $\phi _m^{\text{FF}}(t)$, and rearrange the equation into real and imaginary parts to obtain two equations:

8 $$\begin{aligned} \alpha (t) \text{Im} [ \phi _m^*(\Lambda (t)) \phi _{l}(\Lambda (t))] = \text{Im}\{ \phi _m^*(\Lambda (t)) \phi _l(\Lambda (t)) \exp [i(f_l(t)-f_m(t))]\}, \end{aligned}$$

and

9 $$ \omega _{m}^{{{\text{FF}}}} (t) = {\text{Re}}\left\{ {g\frac{{\phi _{l} (\Lambda (t))}}{{\phi _{m} (\Lambda (t))}}\left( {\alpha (t) - \exp [i(f_{l} (t) - f_{m} (t))]} \right)} \right\} + \alpha (t)\omega _{m} (\Lambda (t)) - \frac{{df_{m} (t)}}{{dt}}, $$

where $l\ne m$. Equation (8) is used to obtain the additional phase $f_m(t)$, and Eq. (9) is used to calculate the time-dependent qubit resonance frequency which yields the speed-controlled dynamics. Note that $f_{m(l)}(t)=0$ is a solution of Eq. (8) when $\alpha =1$. We set $f_1(t)=0$ and consider variations in $f_2(t)$. This is justified given that only the phase difference $f_1(t)-f_2(t)$ is relevant for the dynamics. The above formalism can also be extended to the case in which there exists a tunable coupling *g*(*t*), as shown in Supplementary Sect. S4.

As seen in Eq. (9), the control parameters diverge when $\phi _m(\Lambda (t))$ becomes zero. Because $\phi _m(\Lambda (t))$ is finite in the dynamics considered in this manuscript, we do not encounter divergence of control parameters. If $\phi _m(\Lambda (t))$ becomes zero at particular times $t_i$, the magnification factor $\alpha (t)$ should be designed so that it smoothly becomes unity at $t_i$ to avoid the divergence in the present theory (note that $f_m(t)=0$ is a solution of Eq. (8) when $\alpha (t_i)=1$). Although the development of a method to avoid such divergence for the general case is out of the scope of this paper, we refer readers to the related works for continuous systems, which gives a criterion for determining whether a singularity will arise^17^, and proposes a method to overcome the issue of divergence^15^.

## Acceleration

In order to generate the desired target state from a given initial state, one first must determine the additional phase which vanishes at the initial and final times. However sometimes there exist no such solutions to Eq. (8). Here, we develop the ITT method which realizes the target state approximately in cases where no exact solutions would ordinarily exist.

We consider the acceleration of some reference dynamics, in which $\omega _1$ is decreased for $0\le t \le T$ as

10 $$\begin{aligned} \omega _1(t) =\Delta \omega _{0} \cos (\pi t/ T) + \omega _2, \end{aligned}$$

while $\omega _2$ and *g* are held constant. In Eq. (10), the constant parameter, $\Delta \omega _{0}$, is the value of $\omega _1-\omega _2$ at $t=0$. The time dependence of $\omega _1$ and the population of $|m\rangle$ are shown in Fig. 1b. The wave function of the reference dynamics $\phi _{m}(t)$ is obtained by solving the Schrödinger equation (3) numerically. We consider the acceleration and deceleration of these particular dynamics (the “reference dynamics”) in the following, as this is simply one such case where ITT resolves the shortcomings of FFST. In this example, we set $T=g^{-1}$.

As an example, we use the magnification factor defined by

11 $$\begin{aligned} \alpha (t) = 1 - \frac{T_{\text{F}}-T}{T_{\text{F}}}\bigg \{1 - \cos (2\pi t/T_{\text{F}}) \bigg \}, \end{aligned}$$

where $\alpha$ is chosen to satisfy $\alpha (0) = \alpha (T_{\text{F}})=1$ so that the speed-controlled dynamics coincides with the reference dynamics at $t=0$ and $T_{\text{F}}$. For $T_{\text{F}}<T$ (acceleration), the magnification factor satisfies $\alpha \ge 1$. The time dependence of $\alpha$ is shown in Fig. 1c. In this example, we set $T_{\text{F}}=0.9g^{-1}$, that is, the accelerated dynamics takes 0.9 times less than the reference to reach the desired state.

Figure 1d shows $\beta ^{\text{FF}}$ defined as

12 $$\begin{aligned} \beta ^{\text{FF}}(t,f_2)/g = \alpha (t) \text{Im} [ \phi _1^*(\Lambda (t)) \phi _{2}(\Lambda (t))] - \text{Im}\{ \phi _1^*(\Lambda (t)) \phi _2(\Lambda (t)) \exp [if_2]\}, \end{aligned}$$

which is the difference of the left hand side and the right hand side of Eq. (8) for $f_1(t)=0$. Note that $f_2$ is regarded as a variable in Eq. (12) instead of a solution of Eq. (8). We plot $\beta ^{\text{FF}}(t,f_2)$ only for $-\pi< f_2 < \pi$ given that it is periodic with respect to $f_2$. We note that the zeros of $\beta ^{\text{FF}}(t,f_2)$ correspond to the solutions of Eq. (8).

The trajectories, which are defined by the $f_2(t)$ which satisfy $\beta ^{\text{FF}}(t,f_2(t))=0$, represent the realizable accelerated dynamics. We call these paths “speed-controlled trajectories”. However, in this particular case, there exist no trajectories which can connect the initial state corresponding to $f_2(0)=0$ and the target state $f_2(T_{\text{F}})=0$ given that there are no zeroes of $\beta ^{\text{FF}}(t,f_2)$ around $t=$0.5$g^{-1}$, 0.7$g^{-1}$ and 0.8$g^{-1}$. Thus, the dynamics cannot be accelerated exactly. The mechanism by which gaps between trajectories open for acceleration and deceleration is explained in Supplementary Sect. S5.

**Figure 2:**
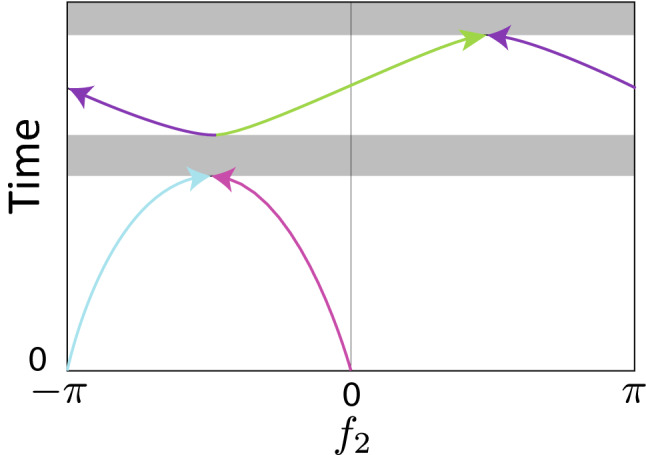
Schematic of viable speed-controlled trajectories in the $f_2-t$ space. Each viable speed-controlled trajectory is represented by a different colored curve. The arrows indicate the direction of time evolution of the solutions, $f_2(t)$. At $t=0$, there are two trivial solutions of $\beta ^{\text{FF}}(t,f_2)=0$, $f_2=0$ and $-\pi$ because $\alpha (0)=1$. In the two time domains marked by gray color, there exists no realizable wave function with the form of Eq. (4).

Now, a comment on viable speed-controlled trajectories is in order. As discussed in Supplementary Sect. S5, $\beta ^{\text{FF}}(t,f_2)=0$ has at most two solutions in $-\pi \le f_2 < \pi$ for each *t*. The dark orange curves (zeros of $\beta ^{\text{FF}}(t,f_2)$) in Fig. 1d represent the time dependence of the solutions, $f_2(t)$. In the time domains when there are two dark curves, there are two sets of the wave function in the form of Eq. (4) and corresponding control parameters, which satisfy the Schrödinger equation (7). Thus, the viable speed-controlled trajectories correspond to the realizable wave function with the form of Eq. (4). Figure 2 schematically represents viable speed-controlled trajectories in the $f_2-t$ space. In the figure, there are two time domains which have two different trajectories, and there are two time domains marked in gray color, where there exists no wave function with the form of Eq. (4) which satisfies the Schrödinger equation (7).

In order to resolve the lack of a continuous path between the initial and final states, we introduce virtual trajectories which allow for navigation across sufficiently shallow gaps. In this paper, virtual trajectories, $f_2(t)$, are chosen so that they exactly or approximately satisfy $f_2(0)=f_2(T_{\text{F}})=0$ and $\beta ^{\text{FF}}(t,f_2(t))\simeq 0$ for all times throughout the system’s evolution, while $f_1(t)=0$. For this example, we set the virtual trajectory to be the same as a viable speed-controlled trajectory for $t\le 0.32 g^{-1}$, that is, $f_2(t)$ satisfies $\beta ^{\text{FF}}(t,f_2(t)) = 0$ exactly for $t\le 0.32 g^{-1}$. For $t>0.32 g^{-1}$, we set the virtual trajectory as $f_2(t)=-\eta _1\exp [-\eta _2(t-\eta _3)^2]$, where $\eta _i$ was chosen to be $\eta _1\simeq 0.124$, $\eta _2\simeq 41.8 g^2$ and $\eta _3\simeq 0.482 g^{-1}$ so that $f_2$ and $df_2/dt$ are continuous for $0\le t\le T_F$ and $|\beta ^{\text{FF}}(t,f_2(t))|$ is small also for $t>0.32 g^{-1}$. The virtual trajectory is shown in Fig. 1d,e. As seen in Fig. 1e, the virtual trajectory is confined to the region where $|\beta ^{\text{FF}}(t,f_2(t))|$ is small. $\omega _m^{\text{FF}}$ can then be calculated for any given virtual trajectory by substituting the corresponding $f_2(t)$ and $f_1(t)=0$ into Eq. (12). While both $\omega _1^{\text{FF}}$ and $\omega _2^{\text{FF}}$ may be time-dependent in general, only the difference between the angular frequencies, $\Delta \omega ^{\text{FF}}=\omega _1^{\text{FF}}-\omega _2^{\text{FF}}$, is of physical importance in this subspace. Thus, only one qubit frequency is required to be tunable, yielding a change to the global phase of the wave function (see Supplementary Sect. S6).

The time dependence of $\Delta \omega ^{\text{FF}}$ and its time derivative corresponding to this virtual trajectory are shown in Fig. 1f,g, respectively. We define the fidelity of the control by the overlap, $|\langle \Psi _{\text{ref}} | \Psi _{\text{ITT}} \rangle |$, between the end state $|\Psi _{\text{ITT}} (T_{\text{F}})\rangle$ of the control with $\omega _m^{\text{FF}}$ and the end state $|\Psi _{\text{ref}} (T)\rangle =\sum _m \phi _m(T)|m\rangle$ of the reference dynamics. The fidelity of the control with ITT is 0.9996 while the fidelity of the control with the naively accelerated control parameters, $\omega _m(\Lambda (t))$, is 0.9871. Therefore, this result shows that ITT can significantly improve the control fidelity compared to a naive scaling of the control parameters with respect to time.

The control with $\alpha (t)\omega _m(\Lambda (t))$, which approximates $\omega ^{\text{FF}}_m$ in Eq. (9), also improves the fidelity compared to the control with $\omega _m(\Lambda (t))$. The fidelity of the control in this case is 0.9989. The improvement of the fidelity for this case is alternatively interpreted as follows. As explained in Supplementary Sect. S4, the ideal dynamics straightforwardly scaled with respect to time is realized if both the coupling strength and angular frequency of the qubits are scaled as $g^{\text{FF}}(t)=\alpha (t) g$, $\omega _m^{\text{FF}}(t)=\alpha (t) \omega _m(\Lambda (t))$. The control with $\alpha (t)\omega _m(\Lambda (t))$ and a fixed coupling strength approximates such dynamics, and thus it also improves the fidelity relative to the naively accelerated control. In experiments, there will be errors in control parameters due to, e.g., the imperfection of their control. The robustness of our method against such errors is examined in Supplementary Sect. S7.

## Inter-trajectory travel for shortcuts to adiabaticity

In this section, we show that ITT can be used to realize shortcuts to adiabaticity. As an example, we consider the case that $\omega _1(t)$ in Eq. (3) is gradually changed while the other parameters are fixed. If $\omega _1(t)$ is changed slowly enough and the initial state is an eigenstate of an initial Hamiltonian, the state remains in the corresponding instantaneous energy eigenstate of the time-dependent Hamiltonian throughout the system’s evolution. On the other hand, rapid changes in $\omega _1(t)$ cause undesired nonadiabatic population transfer to other energy eigenstates and thus increase infidelity. It has been previously shown that FFST can exactly realize the same final state as is produced adiabatically in a time shorter than the adiabatic timescale. In our method, only the detuning is modified in contrast to other methods which require modulation of the coupling^32–34^. However, FFST alone cannot be utilized due to the lack of a viable trajectory when the manipulation time is too short. In the following, we show that ITT can greatly suppress nonadiabatic transitions in such regimes.

**Figure 3:**
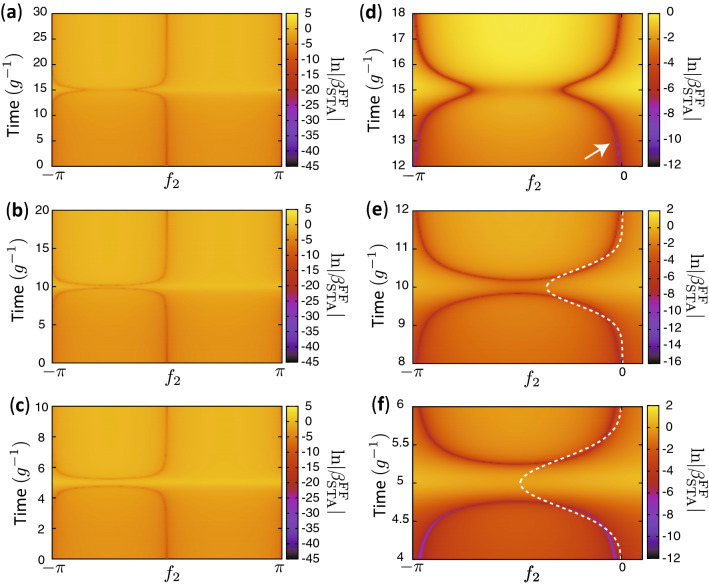
Inter-trajectory travel for shortcuts to adiabaticity. (**a–c**) $\ln |\beta ^{\text{FF}}_{\text{STA}}|$ as a function of $f_2$ and *t*. (**a–c**) are for $T_{\text{F}}=30g^{-1}$, $20g^{-1}$ and $10g^{-1}$, respectively. (**d–f**) are the closeups of panels (**a–c**) respectively. The arrow in (**d**) indicates the speed-controlled trajectory. The white dashed curve in panels (**e,f**) shows a virtual trajectory. $\Delta \omega _0=30g$, where *g* is constant.

We consider some ideal dynamics for which the wave function may be written as $\phi _m(\omega _1(t))e^{-\frac{i}{\hbar }\int _0^t E(\omega _1(t'))dt'}$, where $\phi _m(\omega _1)$ is the wave function of an instantaneous energy eigenstate which satisfies

13 $$\begin{aligned} g \phi _{l}(\omega _1) + \omega _m \phi _{m}(\omega _1) = \frac{E(\omega _1)}{\hbar } \phi _{m}(\omega _1), \end{aligned}$$

where $E(\omega _1)$ is the eigenenergy, and again $m,l\in \{1,2\}$ and $l\ne m$. When $\omega _1(t)$ is changed slowly enough, this is a solution of the Schrödinger equation (3). On the other hand, the state deviates from the expected dynamics when $\omega _1(t)$ is changed on short timescales. Typically, realizing adiabatic dynamics requires a control duration much longer than the time scale given by the energy gap between relevant levels, which is $g^{-1}$ in this example. As shown later, even for the duration of $30 g^{-1}$, there is an apparent unwanted effect due to nonadiabatic transitions. We aim at finding angular frequencies which drive the initial state, $\phi _m(\omega _1(0))$, to the target state, $\phi _m(\omega _1(T_{\text{F}}))e^{-\frac{i}{\hbar }\int _0^{T_{\text{F}}} E(\omega _1(t'))dt'}$, in a short time $T_{\text{F}}$ mitigating such unwanted nonadiabatic transitions.

In FFST one assumes that the wave function of the speed-controlled dynamics has the form

14 $$\begin{aligned} \phi _m^{\text{FF}}(t) = \phi _m(\omega _1(t)) e^{if_m(t)}e^{-\frac{i}{\hbar }\int _0^t E(\omega _1(t'))dt'}. \end{aligned}$$

We assume that $\phi _m^{\text{FF}}$ also satisfies Eq. (7). Using Eqs. (7), (13) and (14), we obtain

15 $$\begin{aligned} \frac{d\phi _m(\omega _1(t))}{dt} = g \phi _l (\omega _1(t)) \sin \bigg [f_l(t) - f_m(t)\bigg ], \end{aligned}$$

and

16 $$\begin{aligned} \omega _m^{\text{FF}}(t) = \omega _m(t) + g\frac{\phi _{l}(\omega _1(t))}{\phi _m(\omega _1(t))} \bigg \{ 1 - \cos \bigg [ f_{l}(t) - f_m(t)\bigg ] \bigg \} - \frac{df_m(t)}{dt}, \end{aligned}$$

where again, $m,l\in \{1,2\}$ and $l\ne m$, we assumed that $\phi _m$ is real. Equation (15) is used to calculate the additional phase, $f_m(t)$, while Eq. (16) is used to calculate the angular frequency, $\omega _m^{\text{FF}}$. We set $f_1(t)=0$ as we did in the previous section. The time dependence of $\omega _1$ is given by

17 $$\begin{aligned} \omega _1(t) = \Delta \omega _0 \cos (\pi t/T_{\text{F}}) + \omega _2, \end{aligned}$$

where $\Delta \omega _0$ is constant, and $T_{\text{F}}$ is the final time of the control.

Figure 3 shows the intensity of $\beta ^{\text{FF}}_{\text{STA}}$ defined as

18 $$\begin{aligned} \beta ^{\text{FF}}_{\text{STA}}(t,f_2) = \frac{d\phi _1(\omega _1(t))}{dt} - g \phi _2 (\omega _1(t)) \sin [f_2], \end{aligned}$$

which is the difference of the left hand side and the right hand side of Eq. (15) for $f_1(t)=0$. For a sufficiently large $T_{\text{F}}$, there exists a viable trajectory which connects the initial state corresponding to $f_2(0)=0$ and the target state $f_2(T_{\text{F}})=0$ as shown in Fig. 3a and 3d for $T_{\text{F}}=30g^{-1}$. $\omega _m^{\text{FF}}(t)$ is obtained using Eq. (16), and $f_2(t)$, which satisfies $\beta ^{\text{FF}}_{\text{STA}}(t,f_2)=0$, corresponds to the speed-controlled trajectory. The obtained $\omega _m^{\text{FF}}(t)$ can realize the target state exactly eliminating the nonadiabatic transition. There are two trajectories because there are two values of $f_2(t)$ which satisfy Eq. (15) in general. One of the trajectories which satisfies $f_2(0)=f_2(T_{\text{F}})=0$ is used to realize the STA. The other trajectory generates different dynamics given a different initial state.

When $T_{\text{F}}$ is not sufficiently long, there is no viable trajectory for the fast-forward protocol as shown in Fig. 3b,c,e,f for $T_{\text{F}}=20g^{-1}$ and $10g^{-1}$, respectively. The vertical gap around $t=T_{\text{F}}/2$ in Fig. 3b,c,e,f is due to the lack of a solution for Eq. (15). We introduce a virtual trajectory which interconnects the two trajectories satisfying $f_2(0) = f_2(T_{\text{F}})\simeq 0$ as represented in Fig. 3e,f. We used a virtual trajectory with the Gaussian form represented as $f_2(t)=-\eta _1\exp [-\eta _2(t-\eta _3)^2]$ for $0\le t \le T_{\text{F}}$. $\eta _1\simeq 0.363$ and $\eta _2\simeq 2.375 g^2$ for Fig. 3e and $\eta _1\simeq 0.495$ and $\eta _2\simeq 5.150 g^2$ for Fig. 3f, while $\eta _3=T_{\text{F}}/2$ for both Fig. 3e,f. The values of $\eta _{1,2}$ were determined so that $\int _0^{T_{\text{F}}}|\beta _{\text{STA}}^{\text{FF}}(t,f_2(t))| dt$ is minimized. The time-dependent frequency $\omega _m^{\text{FF}}(t)$ calculated with these virtual trajectories can realize the target state approximately.

**Figure 4:**
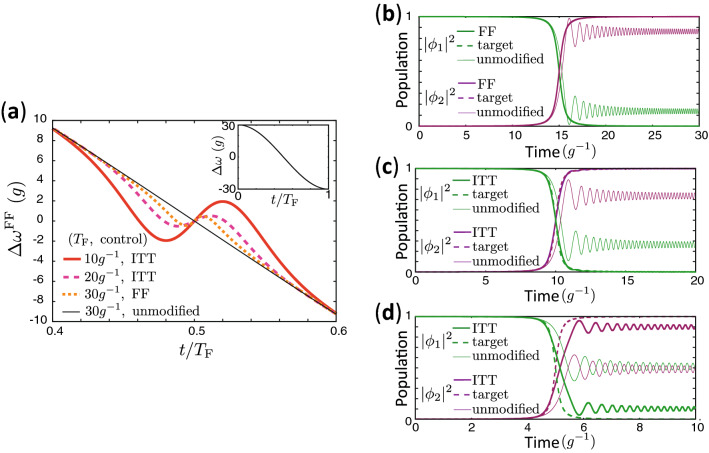
Time dependence of qubit resonance frequency and populations. (**a**) Time dependence of $\Delta \omega$ for the unmodified control with $T_{\text{F}}=30g^{-1}$ (thin black solid curve), $\Delta \omega ^{\text{FF}}$ for FFST with $T_{\text{F}}=30g^{-1}$ (orange dotted curve), $\Delta \omega ^{\text{FF}}$ for ITT with $T_{\text{F}}=20g^{-1}$ (pink dashed curve) and $T_{\text{F}}=10g^{-1}$ (red solid curve). (**b**) Time dependence of the population of $|m\rangle$ in the unmodified control and the control with FFST for $T_{\text{F}} = 30g^{-1}$. The corresponding speed-controlled trajectory is shown in Fig. 3a,d. The population of $|m\rangle$ in the target dynamics, $|\phi _m(\omega _m(t))|^2$, is also shown. The curves for the target dynamics are overlapping with the ones for FFST. (**c,d**) Time dependence of the population of $|m\rangle$ for the unmodified control and ITT in Fig. 3e,f. The populations in the target dynamics are also shown.

We compare the results of FFST and ITT with the unmodified control which utilizes the unmodified angular frequency, $\omega _1(t)$, in Eq. (17). Figure 4a shows the time dependence of the difference between the angular frequencies for the unmodified control, FFST, and ITT. It is seen that the angular frequencies are most drastically adjusted at the halfway point of evolution around $t=T_{\text{F}}/2$ when the wave function radically changes. The modification becomes larger as $T_{\text{F}}$ is made shorter corresponding to a widening of the gap between trajectories.

Figure 4b–d show the time dependence of the population of $|m\rangle$ for the unmodified control, the controls with FFST, and with ITT. In the target dynamics, $|\phi _1|^2\simeq 0$ and $|\phi _2|^2\simeq 1$ at $t=T_{\text{F}}$. On the other hand, $|\phi _1|^2$ and $|\phi _2|^2$ deviate from their desired populations at $t=T_{\text{F}}$ for the dynamics defined by the trajectory where $f_2(t)=0$ at all times, due to unwanted nonadiabatic effects. Figure 4b shows that FFST realizes the exact target dynamics, while the fidelity, which is defined by the overlap with the target state at $t=T_{\text{F}}$, for the unmodified control is 0.929. Figure 4c,d show that ITT can suppress nonadiabatic contributions and faithfully realize the approximate target state. The fidelities of the controls are 0.999 and 0.949 for $T_{\text{F}}=20g^{-1}$ and $T_{\text{F}}=10g^{-1}$ with ITT, while the fidelities are 0.857 and 0.697 for the unmodified controls with $T_{\text{F}}=20g^{-1}$ and $T_{\text{F}}=10g^{-1}$, respectively. The fidelity when utilizing ITT is considerably higher than that of the unmodified controls, although the efficiency of ITT is also degraded as $T_{\text{F}}$ becomes shorter due to the gap between the speed-controlled trajectories widening as seen in Fig. 3f. The robustness of our method against errors in control parameters is examined in Supplementary Sect. S7.

In this section, we considered the case that the coupling strength, *g*, is fixed. We emphasize that FFST and ITT based on FFST can easily take into account such a constraint of the Hamiltonian because the constraint can be included in the beginning of the theory, in contrast to other STA protocols such as counter-diabatic (CD)^35^ and invariant-based inverse engineering (IIE) protocols using Lewis-Riesenfeld (LR) invariants^34^. Another advantage of our protocol is that the theory can be systematically extended to *N*-level systems without finding a nontrivial invariant of dynamics (See Supplementary Sect. S8 for the detail). The reason why FFST cannot generate the target state exactly when the control duration is short, is that there is the constraint of the Hamiltonian. On the other hand, CD protocols directly seek out the Hamiltonian which exactly generates predetermined adiabatic dynamics, and therefore the driving Hamiltonian does not satisfy the constraint of the Hamiltonian in general. In the IIE using LR invariants, firstly an invariant of the dynamics is looked for so that the corresponding driving Hamiltonian matches to the desired ones at the initial and final times. However, constraints for the intermediate Hamiltonian are not imposed usually. For example, STA was studied for a similar two-level system using IIE^34^. The control requires modification both of the diagonal and off-diagonal elements, which correspond to the control parameters and coupling strength, respectively.

## Deceleration based on inter-trajectory travel

We next consider deceleration of the reference dynamics based on ITT. We use the same form of the magnification factor, $\alpha (t)$, as Eq. (11) with $T_{\text{F}}>T$ such that $0 < \alpha (t) \le 1$ is satisfied for the decelerated dynamics. We set $T=g^{-1}$.

**Figure 5:**
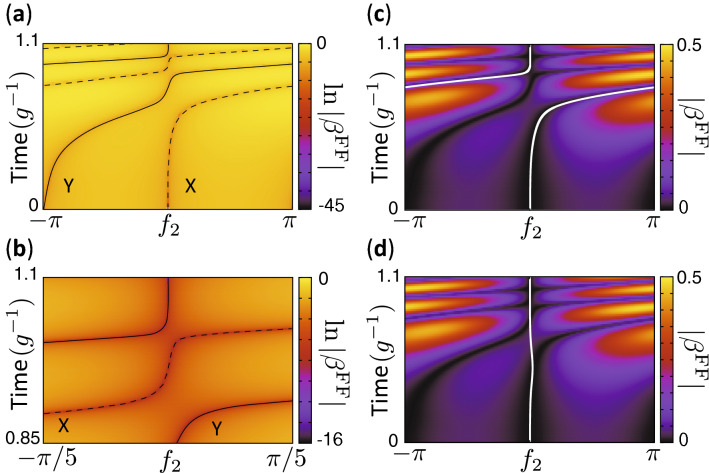
Speed-controlled and virtual trajectories for deceleration. (**a**) $\ln |\beta ^{\text{FF}}|$ as a function of $f_2$ and *t* for $T_{\text{F}}=1.1g^{-1}$. Other parameters used are the same as in Fig. 1b. The dashed and solid curves indicate SCT-X and SCT-Y. The dashed line does not reach $f_2=0$ at $t=1.1g^{-1}$. (**b**) A closeup of (**a**). (**c**) VT-A indicated by the white curve interpolating the speed-controlled trajectories at around $t=0.9g^{-1}$. (**d**), VT-B interpolating the speed-controlled trajectories at around $t=0.7g^{-1}$ and 0.9$g^{-1}$. The color in (**c,d**) shows $|\beta ^{\text{FF}}|$ as a function of $f_2$ and t.

Figure 5a,b shows $\beta ^{\text{FF}}$ as a function of $f_2$ and *t* for $f_1=0$ and $T_{\text{F}}=1.1g^{-1}$. In this example, the decelerated dynamics takes 1.1 times longer than the unmodified dynamics to reach the desired state. For the parameters we consider, there are two speed-controlled trajectories, X and Y (SCT-X and SCT-Y), as represented in Fig. 5a,b given that there are two possible sets of values for $f_2(t)$ which satisfy $\beta ^{\text{FF}}(t,f_2)=0$. As seen in Fig. 5a,b, there are narrow gaps between the speed-controlled trajectories around $t=0.7g^{-1},~0.9g^{-1}$ and $g^{-1}$. Importantly, there exist no trajectories which can connect the initial state corresponding to $f_2(0)=0$ and the target state $f_2(T_{\text{F}})=0$.

As shown previously for the case of accelerated dynamics, ITT can also approximately realize the desired end state for decelerated dynamics. We consider two of the possible virtual trajectories in this study. The virtual trajectories are shown in Fig. 5c (virtual trajectory A [VT-A]) and Fig. 5d (virtual trajectory B [VT-B]). For VT-A, we set the virtual trajectory to be the same as a viable speed-controlled trajectory for $t\le 0.9585 g^{-1}$, and set the virtual trajectory as $f_2(t)=0$ for $t> 0.9585 g^{-1}$. For VT-B in Fig. 5d, we set the virtual trajectory to be the same as a viable speed-controlled trajectory for $t\le 0.3439 g^{-1}$, and set the virtual trajectory as $f_2(t)=-\eta _1\exp [-\eta _2(t-\eta _3)^2]$ for $t>0.3439 g^{-1}$, where $\eta _i$ was chosen to be $\eta _1\simeq 0.0646$, $\eta _2\simeq 39.2 g^2$ and $\eta _3\simeq 0.497 g^{-1}$ so that $f_2$ and $df_2/dt$ are continuous for $0\le t\le T_F$ and $|\beta ^{\text{FF}}(t,f_2(t))|$ is small also for $t>0.3439 g^{-1}$. Both the virtual trajectories satisfy exactly or approximately $f_2(0)=f_2(T_{\text{F}})=0$ and $\beta ^{\text{FF}}(t,f_2(t))\simeq 0$ for all times. $f_2=\pi$ and $-\pi$ are regarded as the same point given that $\beta ^{\text{FF}}(t,f_2)$ is periodic with respect to $f_2$. Thus, VT-A is also continuous, although there is a jump from $\pi$ to $-\pi$ in Fig. 5c. We show in the following that the state of the system can approximately trace a selected virtual trajectory, although the virtual trajectory is not an exact solution of the Schrödinger equation.

**Figure 6:**
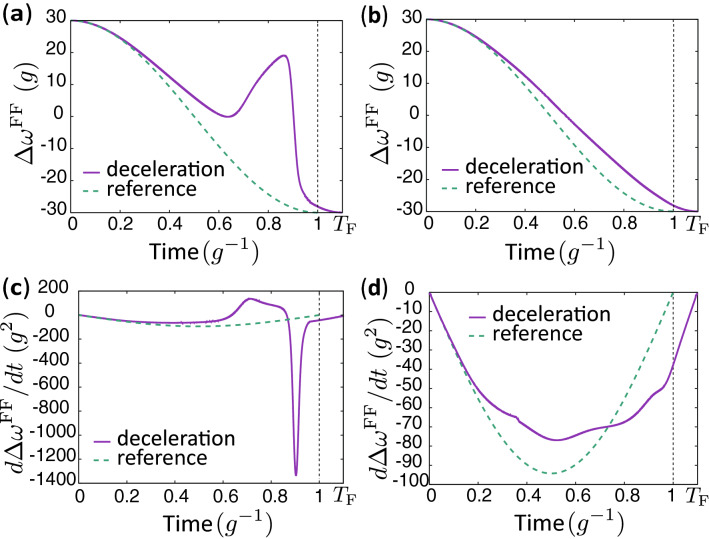
Difference of qubit frequencies and its time derivative. (**a,b**) Time dependence of $\Delta \omega ^{\text{FF}}=\omega _1^{\text{FF}}-\omega _2^{\text{FF}}$ for the virtual trajectories. The dashed curve represents the $\Delta \omega =\omega _1-\omega _2$ used in the reference dynamics. The parameters used are the same as in Fig. 5. (**c,d**) Time dependence of $d\Delta \omega ^{\text{FF}}/dt$ and $d\Delta \omega /dt$. (**a,c**) are for virtual trajectory A, and (**b,d**) are for virtual trajectory B.

Figure 6 shows the time dependence of $\Delta \omega ^{\text{FF}}$ and its time derivative for both trajectories. The time dependence of $\Delta \omega ^{\text{FF}}$ for VT-A is complicated compared to the one for VT-B due to the rate of change of $f_2(t)$ for VT-A being more rapid than for VT-B. The fidelity of the control is 0.99998 and 0.9995 for VT-A and VT-B, respectively, while the fidelity of the control with the naively decelerated control parameters, $\omega _m(\Lambda (t))$, is 0.9876. The fidelity of the control with $\alpha (t)\omega _m(\Lambda (t))$ is 0.9984.

**Figure 7:**
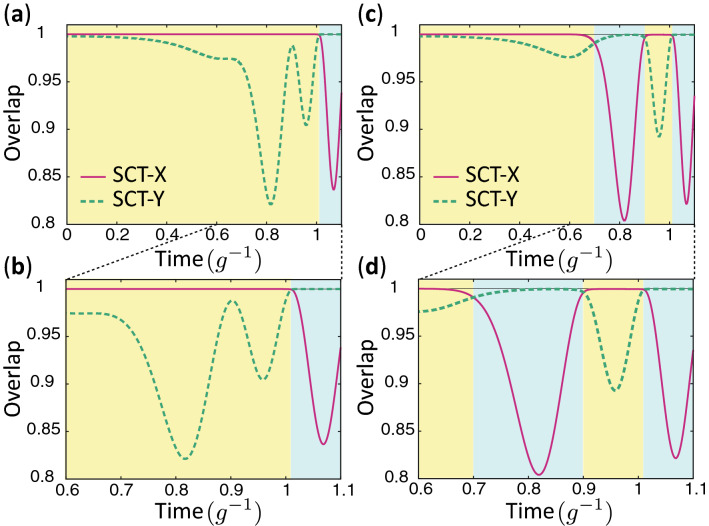
Overlaps with the speed-controlled trajectories. (**a,b**) are for ITT A and (**c,d**) are for ITT B. In the yellow region, the overlap with the SCT-X is larger than the one with the SCT-Y, while the overlap with the SCT-Y is larger than the one with the SCT-X in the blue region.

Figure 7 illustrates the shifts between viable speed-controlled trajectories $|\Psi ^{\text{FF}}_{X/Y}(t)\rangle =\sum _m\phi _{m,X/Y}^{\text{FF}}(t)|m\rangle$ that occur while a state follows a virtual trajectory given by $|\Psi _{\text{ITT}} (t)\rangle$, where $\phi _{m,X/Y}^{\text{FF}}(t)$ is defined by Eq. (4) with $f_m(t)$ corresponding to each speed-controlled trajectory. VT-A initially starts from SCT-X and approximately traces it, and near the end of its evolution shifts to SCT-Y as shown in Fig. 7a. In the yellow region, the overlap with the trajectory X is greater than with the trajectory Y. In the light blue region, the overlap with trajectory Y is dominant. Thus, this result indicates the occurrence of trajectory shifts. An ITT event occurs once for VT-A and three times for VT-B as shown in Fig. 7a and b, respectively.

## Methods

We numerically simulated the dynamics of the system composed of two coupled qubits to evaluate the efficiency of our control protocols. We considered the case that the dynamics of the system is confined to a subspace spanned by two states, and numerically solved the time-dependent Schrödinger equation, presented e.g. in Eq. (3), with a fourth-order Runge-Kutta integrator with time steps of $5 \times 10^{-8} g^{-1}$. The generated data are included in the supplementary information files.

## Conclusions

We have developed a novel method for the acceleration and deceleration of quantum dynamics, which we call inter-trajectory travel (ITT). ITT is based on the knowledge of the structure of speed-controlled trajectories and gaps between those trajectories. A virtual trajectory interconnecting different speed-controlled trajectories enables one to derive control parameters which either accelerate or decelerate the dynamics of a quantum system. Our method has extended the applicability of FFST by overcoming the non-existence of viable trajectories in the existing theory. Furthermore, we have applied ITT to the study of shortcuts to adiabaticity and successfully shown that the same target state can be realized in a shorter time when compared to the adiabatic dynamics by suppressing unwanted nonadiabatic transitions. The acceleration of quantum dynamics via ITT provides a novel way to outrun decoherence effects when manipulating quantum dynamics by solely modifying qubit frequencies.

We have also shown that the application of ITT for deceleration can be used to find slower control parameters which generate approximately the same target state. We consider ITT to be useful for state preparation with modern quantum technologies as it allows one to design control parameters so that they may satisfy experimental limitations in laboratory control hardware by loosening the often strict requirement of rapid and precise variation of parameters.

An advantage of ITT is that it does not require iterative integrations of equations of motion in contrast to trial & error protocols such as quantum optimal control theories. Importantly, our method is complementary with other protocols. For example, our method can be used to modify the speed of the dynamics derived by other protocols in order to make the control parameters more experimentally feasible or to make the control duration shorter. Thus, our technique adds to the quantum control toolbox which experimentalists may draw from to determine optimal parameters^36–42^.

## Supplementary Information

Supplementary Information.

## Data Availability

All data generated or analysed during this study are included in this published article and its Supplementary Information files.
