# Supplementary material for: Acceleration and deceleration of quantum dynamics based on inter-trajectory travel with fast-forward scaling theory
Source: Sci Rep. 2022 Jun 24;12:10744. doi: 10.1038/s41598-022-14973-6 (PMC9232509; doi:10.1038/s41598-022-14973-6)
Supplement: Supplementary file 1 — Supplementary Information. [file 41598_2022_14973_MOESM1_ESM.pdf]

Supplemental information: Acceleration and deceleration of quantum dynamics based on inter-trajectory travel with fast-forward scaling theory

Shumpei Masuda[1,*], Jacob Koenig[2,†] and Gary A. Steele[2]

[1] Research Center for Emerging Computing Technologies (RCECT), National Institute of Advanced Industrial Science and Technology (AIST), 1-1-1, Umezono, Tsukuba, Ibaraki 305-8568, Japan
[2] Kavli Institute of Nanoscience, Delft University of Technology, Lorentzweg 1, 2628 CJ, Delft, The Netherlands

[*] shumpei.masuda@aist.go.jp
[†] j.d.koenig@tudelft.nl

S1 Realization with superconducting qubits

We consider the case of two superconducting transmon qubits with a fixed capacitive coupling g far less than the resonance frequency of either qubit. The Hamiltonian of the system can be written as

$$H = \omega_1(t) a_1^\dagger a_1 + \frac{\alpha_1}{2} a_1^\dagger a_1^\dagger a_1 a_1 + \omega_2 a_2^\dagger a_2 + \frac{\alpha_2}{2} a_2^\dagger a_2^\dagger a_2 a_2 + g(a_1^\dagger a_2 + a_2^\dagger a_1), \tag{S1}$$

where a_i and α_i are the annihilation operator and anharmonicity parameter of qubit i and we work in units where $\hbar = 1$. The annihilation operator can be represented as $a_i = \sum_{n=1} \sqrt{n} |n-1\rangle_i \langle n|_i$, where n is an integer and $|n\rangle_i$ is a Fock state of qubit i. We have $[a_i, a_i^\dagger] = a_i a_i^\dagger - a_i^\dagger a_i = 1$. Each qubit's resonance frequency and the coupling between them may be written as

$$\omega_i = \sqrt{8E_{Ji}E_{Ci}} - E_{Ci} \tag{S2}$$

$$g = \frac{E_{Cc}}{\sqrt{2}} \left(\frac{E_{J1}E_{J2}}{E_{C1}E_{C2}}\right)^{1/4} \tag{S3}$$

provided $E_{Ji} \gg E_{Ci}$, where E_{Ji} and $E_{Ci} \approx -\alpha_i$ are the Josephson and charging energies of qubit i respectively, with E_{Cc} the charging energy of the coupling capacitor[1,2]. Usually, qubits are designed so that ω_i is much larger than α_i and g[4]. In general, the role of the anharmonicity α_i is to make uneven the intervals between energy levels of a qubit so that the unwanted population transfers to higher levels, $|n(\geq 2)\rangle_i$, are reduced, when the qubit is driven by a resonant microwave pulse[4] (a microwave pulse is not used in our control). When g is sufficiently smaller than ω_i, mixing between higher levels $|n(\geq 2)\rangle_i$ and qubit states due to the coupling is negligible, because higher levels are energetically separated well from the states in the qubit subspace. Then, we may truncate $|n(\geq 2)\rangle_i$ in the above Hamiltonian and obtain Eq. (1). In the truncation, we replace a_i by $\sigma_i = |0\rangle_i\langle 1|_i$, where σ_i satisfies $\{\sigma_i, \sigma_i^\dagger\} = \sigma_i\sigma_i^\dagger + \sigma_i^\dagger\sigma_i = 1$. To realize the scheme outlined in the main text, the most straightforward approach is to use one tunable-frequency qubit with resonance frequency $\omega_1(t)$ and one fixed-frequency qubit with ω_2. We consider an asymmetric transmon for which the two Josephson junctions which comprise its SQUID loop have different Josephson energies. The frequency of qubit 1 is tunable by varying the applied flux Φ through the loop, given that

$$E_{J1}(\Phi) = E_{J1}^{\max} \cos\left(\frac{\pi\Phi}{\Phi_0}\right) \sqrt{1 + d^2 \tan^2\left(\frac{\pi\Phi}{\Phi_0}\right)}, \tag{S4}$$

where $E_{J1}^{\max}$ is the total, maximum Josephson energy of the loop, Φ_0 is the magnetic flux quantum, and d is a measure of the junction asymmetry[3]. The applied flux Φ may be varied in time to satisfy the requirements on the time-dependent frequency. As an example, when considering the accelerated dynamics in Fig. 1f and the decelerated dynamics in Fig. 6b, the flux should be smoothly tuned from $\Phi(0) = 0$ to $\Phi(T_F) = 0.5\Phi_0$ in a time $T_F = 0.9g^{-1}$ or $T_F = 1.1g^{-1}$. As one particular example, the accelerated and decelerated dynamics can be closely replicated for a total evolution time on the order of 10^2ns for $E_{J1}^{\max} =$ 30GHz, $E_{J2} = 27.7$GHz, $E_{C1,2} = 203$MHz, $E_{Cc} = 107$MHz, $g/2\pi = 9$MHz, and $d = 0.85$, which is comparable to modern gate times with transmon qubits and far shorter than standard relaxation and dephasing times typically on the order of tens of microseconds.

In the section entitled "Inter-trajectory travel for shortcuts to adiabaticity", a longer $T_{\rm F}$ is used. The largest value of $T_{\rm F}$ used in this study is $T_{\rm F} = 30g^{-1}$. For the control with large $T_{\rm F}$, we can chose a larger value of g so that the control duration is sufficiently smaller than the coherence time. We can make g larger, e.g., by making the coupling capacitance characterized by E_{Cc} larger. For example, we have $30g^{-1} = 0.16\ \mu$s for $g/2\pi = 30$ MHz.

S2 Single qubit

In this section, we show that the dynamics examined in the main text can be emulated by a superconducting transmon qubit under a drive field. The Hamiltonian of the system is written as

$$\frac{H}{\hbar} = \omega(t)a^\dagger a + \frac{\alpha_0}{2}a^\dagger a^\dagger aa + 2\Omega\cos(\omega_{\rm d}t)(a+a^\dagger), \tag{S5}$$

where ω and α_0 are the angular frequency and anharmonicity parameter of the transmon, and Ω and $\omega_{\rm d}$ are the Rabi frequency and angular frequency of the drive field. Now we move to a rotating frame with frequency ω_d and use the rotating wave approximation (RWA) to obtain

$$\frac{H_{\rm RWA}}{\hbar} = \Delta(t)a^\dagger a + \frac{\alpha_0}{2}a^\dagger a^\dagger aa + \Omega(a+a^\dagger), \tag{S6}$$

where $\Delta(t) = \omega(t) - \omega_{\rm d}$. We assume that $|\Delta|, \Omega \ll |\alpha_0|$. Then the system can be approximated as a two-level system for which the Hamiltonian is represented as

$$H_{\rm RWA} = \frac{\hbar\Delta}{2}\sigma_z + \hbar\Omega\sigma_x, \tag{S7}$$

where we shifted the origin of the energy by $\hbar\Delta/2$. This is effectively the same as the system for which the dynamics is governed by Eq. (3). Therefore, the accelerated and decelerated dynamics examined in the main text can be emulated by this single qubit system under a drive, although the detuning should be sufficiently smaller than α_0.

S3 Role of additional phase

We discuss the role of additional phase by showing how the relative phase between ϕ_m and ϕ_l influences the time dependence of the population. Using Eq. (3), the time derivative of the population $|\phi_m|^2$ can be written as

$$\frac{d}{dt}|\phi_m|^2 = -ig\phi_m^*\phi_l + ig\phi_m\phi_l^*. \tag{S8}$$

Equation (S8) can be rewritten as

$$\frac{d}{dt}|\phi_m|^2 = -2g\tilde{\phi}_m\tilde{\phi}_l\sin(\theta_m - \theta_l), \tag{S9}$$

where $\tilde{\phi}_m$ and θ_m are the intensity and the phase of ϕ_m, that is, $\phi_m = \tilde{\phi}_m e^{i\theta_m}$ where $\tilde{\phi}_m, \theta_m \in R$.

Thus, it is seen that the relative phase $\theta_m - \theta_l$ affects the rate of change of the population as well as the intensity $\tilde{\phi}_m$ and coupling g. The intensity of the wave function and the coupling of the accelerated or decelerated dynamics are the same as in the reference dynamics in our formalism. Therefore, deformation of the phase via additional phase is required for acceleration and deceleration.

S4 Tunable coupling

The fast-forward scaling theory can be extended straightforwardly to the case in which the coupling strength is tunable. The reference dynamics develops under time-dependent g and ω_m. The Schrödinger equation is represented by

$$i\frac{d}{dt}\phi_m(t) = g(t)\phi_l(t) + \omega_m(t)\phi_m(t). \tag{S10}$$

We extend the formalism to obtain g and ω_m which accelerate, decelerate, or reverse the system evolution relative to the reference dynamics. If g and ω_m can be perfectly controlled, we can use a trivial scaling property as explained later. However, the controllability of the parameters is limited in speed and range by device parameters and control hardware. Therefore, it will be meaningful to develop fast-forward scaling theory also for the case with a tunable coupling as the theory would then provide various ways to generate a target state.

We assume that the wave function, $\phi_m^{\rm FF}(t)$, of the accelerated, decelerated, or reversed dynamics has the same form as Eq. (4). We assume that $\phi_m^{\rm FF}$ is a solution of the Schrödinger equation:

$$i\frac{d}{dt}\phi_m^{\rm FF}(t) = g_{\rm FF}(t)\phi_l^{\rm FF}(t) + \omega_m^{\rm FF}(t)\phi_m^{\rm FF}(t). \tag{S11}$$

In the same manner as used for Eqs. (8) and (9), we can obtain two equations:

$$\alpha(t)g(t)\mathrm{Im}[\phi_m^*\phi_l] = g^{\mathrm{FF}}(t)\mathrm{Im}\{\phi_m^*\phi_l\exp[i(f_l - f_m)]\} \tag{S12}$$

and

$$\omega_m^{\mathrm{FF}}(t) = \mathrm{Re}\left\{\frac{\phi_l}{\phi_m}\Big(\alpha(t)g(t) - g^{\mathrm{FF}}(t)\exp[i(f_l - f_m)]\Big)\right\} + \alpha(t)\omega_m(\Lambda(t)) - \frac{df_m}{dt}, \tag{S13}$$

where $l \neq m$, and $\phi_{m(l)}$ and $f_{m(l)}$ abbreviate $\phi_{m(l)}(\Lambda(t))$ and $f_{m(l)}(t)$, respectively.

Equations (S12) and (S13) have a trivial solution $g^{\mathrm{FF}}(t) = \alpha(t)g(t)$, $\omega_m^{\mathrm{FF}}(t) = \alpha(t)\omega_m(\Lambda(t))$ and $f_m(t) = 0$. The corresponding dynamics is simply scaled with respect to time without any additional phase. However, in general, $g^{\mathrm{FF}}(t)$ can be chosen to be different from $\alpha(t)g(t)$. Thus, Eqs. (S12) and (S13), which encompass the simply scaled dynamics, provide us with various choices for the time dependence of the control parameters for acceleration, deceleration, or reversal.

S5 Gaps between trajectories

In order to examine the mechanism by which the gaps between trajectories manifest, we rewrite Eq. (8) as

$$\alpha(t)b(t) = b(t)\cos(f_l(t) - f_m(t)) + a(t)\sin(f_l(t) - f_m(t)), \tag{S14}$$

where $a(t) = \mathrm{Re}[\phi_m^*\phi_l]$ and $b(t) = \mathrm{Im}[\phi_m^*\phi_l]$, and $\phi_{m(l)}$ abbreviates $\phi_{m(l)}(\Lambda(t))$ in this section. Equation (S14) can be rewritten as

$$\frac{\alpha(t)b(t)}{r(t)} = \sin(f_l(t) - f_m(t) + \theta(t)), \tag{S15}$$

where $r(t) = \sqrt{a(t)^2 + b(t)^2}$ and $\tan\theta(t) = b(t)/a(t)$. Equation (S15) has at most two solutions of $f_l(t) - f_m(t)$ for $-\pi \leq f_l(t) - f_m(t) < \pi$. When $\phi_m^*\phi_l$ is purely imaginary $a(t) = 0$, $b(t) \neq 0$, and $\alpha(t) = 1$, two solutions are degenerate at $f_l - f_m = 0$.

Figure S1 shows $\ln|\beta^{\mathrm{FF}}|$ for various values of T_{F} for $f_1(t) = 0$. In Fig. S1a for $T_{\mathrm{F}} = g^{-1}$, there is a trivial trajectory at $f_2 = 0$ given that $\alpha(t) = 1$. This trajectory corresponds to the reference dynamics. The intersections of the trajectories at $f_2 = 0$ in Fig. S1a correspond to the degeneration points where $\phi_m^*\phi_l$ is purely imaginary. The intersections are disconnected and the gap between the trajectories opens in Fig. S1b–e where $\alpha(t) \neq 1$ for $0 < t < T_{\mathrm{F}}$. The gap between the trajectories opens horizontally for the decelerated dynamics as seen in Fig. S1b and S1c which correspond to $\alpha(t) < 1$. On the other hand, the gap between the trajectories opens vertically for the acceleration as seen in Fig. S1d and S1e which correspond to $\alpha(t) > 1$. This is because there are two solutions of $f_2(t)$ for $\alpha(t) < 1$, while there is no solution of $f_2(t)$ for $\alpha(t) > 1$, when $\phi_m^*\phi_l$ is purely imaginary.

S6 Shift of ω

We show that differences between $\omega_1(t)$ and $\omega_2(t)$ give rise to changes in the overall phase of the wave function. We assume that ϕ_m satisfies Eq. (3). Now, we introduce $\bar{\phi}_m(t)$ defined by

$$\bar{\phi}_m(t) = \phi_m(t)e^{i\theta(t)}, \tag{S16}$$

where $\theta(t)$ is independent of m. Using Eq. (3), we obtain

$$i\frac{d}{dt}\bar{\phi}_m(t) = g\bar{\phi}_l(t) + \big(\omega_m(t) - \dot{\theta}(t)\big)\bar{\phi}_m(t), \tag{S17}$$

This result represents that the wave function simply acquires additional global phase when $\omega_1(t)$ and $\omega_2(t)$ are shifted by the same amount, $-\dot{\theta}(t)$.

S7 Robustness

We examine the robustness of our method against errors in control parameters for the control discussed in the section entitled “Acceleration". We consider the control parameters with error, defined by

$$\overline{\omega}_m^{\mathrm{FF}}(t) = \xi[\omega_m^{\mathrm{FF}}(t) - \omega_m(\Lambda(t))] + \omega_m(\Lambda(t)), \tag{S18}$$

where ξ is a parameter which characterizes the accuracy of the modification of the control parameters. In Eq. (S18), the last term represents the control parameter for the unmodified control, while the first term proportional to ξ represents the

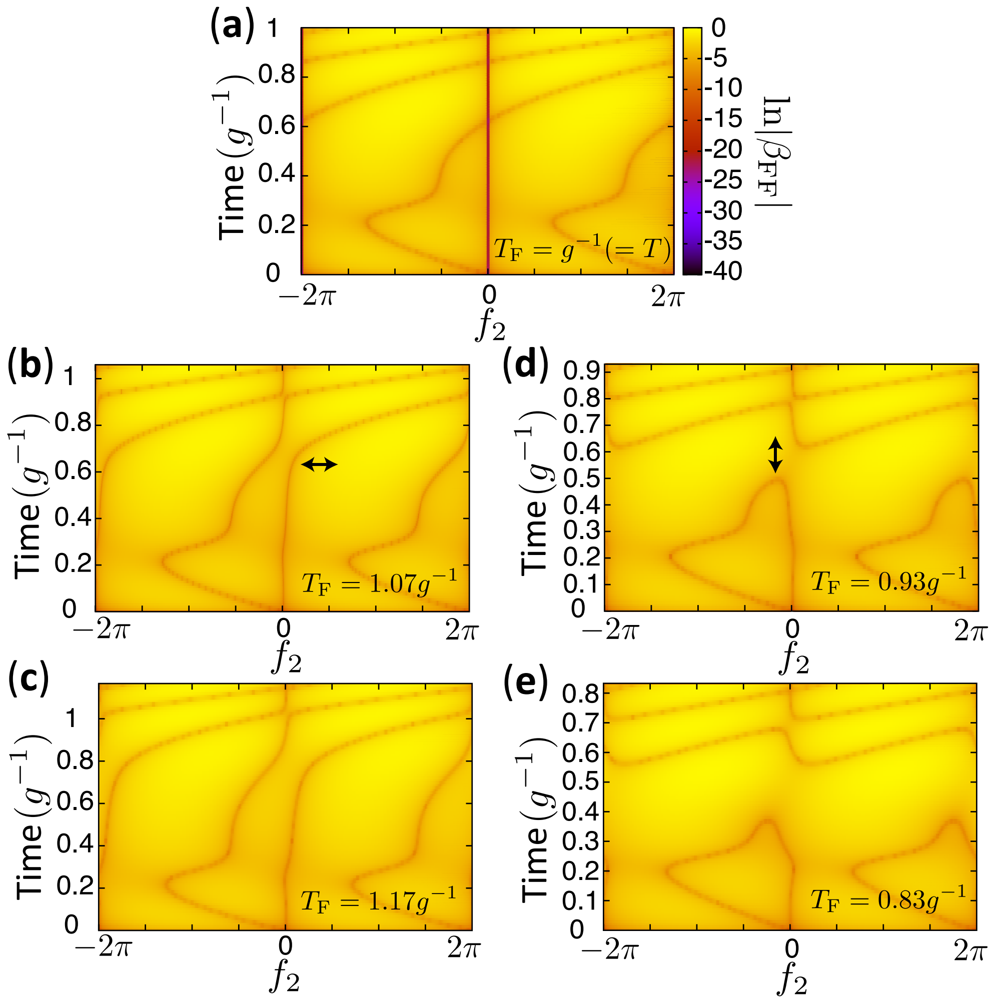

Figure S1. Gap opening between speed-controlled trajectories. (a)–(e), $\ln|\beta^{\rm FF}|$ as a function of f_2 and t for $T_{\rm F}$ indicated in the panels. Other parameters used are the same as in Fig. 1b. On the brown lines, $f_2 = 0$ and $f_2 = \pm 2\pi$, in (a), $\beta^{\rm FF}(t, f_2) = 0$ ($\ln|\beta^{\rm FF}(t, f_2)| = -\infty$). These lines correspond to the reference dynamics. (b),(c) correspond to deceleration, and (d),(e) to acceleration. The arrows in (b) and (d) represent the direction in which the gap between the trajectories opens.

auxiliary part introduced for the speed control. For $\xi = 1$, the control parameters do not have any errors. For $\xi < (>) 1$, the auxiliary part of the control parameters is too small (large). For $\xi = 0$, there is no auxiliary part, and thus $\overline{\omega}_m^{\rm FF}(t)$ coincides with $\omega_m(\Lambda(t))$, and the control is identical to the unmodified control. Figure S2 shows the dependence of the fidelity of the control on ξ. It is seen that the fidelity is higher than the one for the unmodified control in the range of ξ considered here.

In an analogous manner, we examine the robustness for the control discussed in the section entitled "Inter-trajectory travel for shortcuts to adiabaticity". We consider the control parameters with error defined by

$$\overline{\omega}_m^{\rm FF}(t) = \xi[\omega_m^{\rm FF}(t) - \omega_m(t)] + \omega_m(t). \tag{S19}$$

As explained above, ξ is a parameter characterizing the accuracy of the control parameters. Figure S3 shows the dependence of the fidelity of the control on ξ. The fidelity is higher than the one of the unmodified control in the range of ξ considered here.

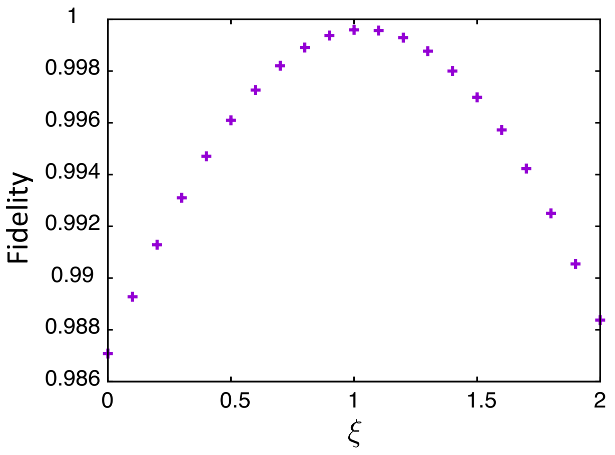

Figure S2. Dependence of fidelity on ξ in Eq. (S18) for the control discussed in the section entitled "Acceleration". ξ is a parameter characterizing the accuracy of the control parameters. The control parameters do not have an error for $\xi = 1$; the control is identical to the unmodified control for $\xi = 0$. The other used parameters are the same as in Fig. 1.

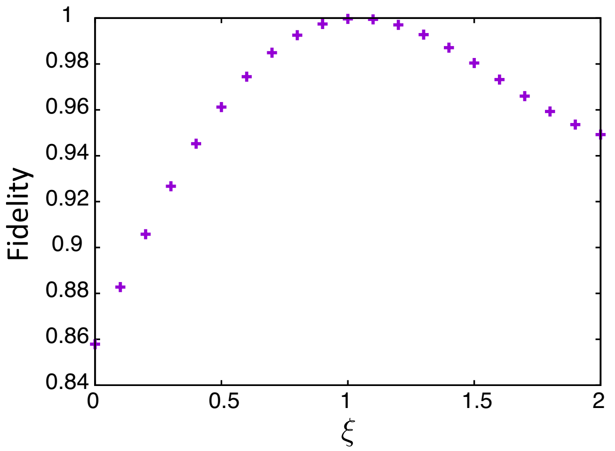

Figure S3. Dependence of fidelity on ξ in Eq. (S19) for the control discussed in the section entitled "Inter-trajectory travel for shortcuts to adiabaticity" wtih $T_{\mathrm{F}} = 20g^{-1}$. ξ is a parameter characterizing the accuracy of the control parameters. The control parameters do not have an error for $\xi = 1$; the control is identical to the unmodified control for $\xi = 0$. The other parameters are the same as in Fig. 3.

S8 Multi-level systems

ITT can be applied to multi-level systems with more than two levels. In this section, we show ITT for shortcuts to adiabaticity in multi-level systems. We consider an N-level system for which the Schrödinger equation is written as

$$i\hbar\frac{d\Psi_m(t)}{dt} = \sum_{l(\neq m)}^{N} g_{ml}\Psi_l(t) + \omega_m(t)\Psi_m(t), \tag{S20}$$

where $\Psi_m(t)$ is the wave function corresponding to the mth level. In Eq. (S20), g_{ml} is assumed to be constant for simplicity. If the control parameters $\vec{\omega} = \{\omega_1, \omega_2, \cdots, \omega_N\}$ are changed slowly enough and if the initial state is in an energy eigenstate, the system remains in the same instantaneous energy eigenstate, and the wave function is written as $\Psi_m(t) = \phi_m(\vec{\omega}(t))e^{-\frac{i}{\hbar}\int_0^t E(\vec{\omega}(t'))dt'}$. Here, $\phi_m(\vec{\omega})$ is the wave function of an instantaneous energy eigenstate, which satisfies the time-independent Schrödinger equation,

$$\sum_{l(\neq m)}^{N} g_{ml}\phi_l(\vec{\omega}) + \omega_m\phi_m(\vec{\omega}) = \frac{E(\vec{\omega})}{\hbar}\phi_m(\vec{\omega}), \tag{S21}$$

and $E(\vec{\omega})$ is the eigenenergy. We aim at finding the control parameters which drive the initial state, $\phi_m(\vec{\omega}(0))$, to the target state, $\phi_m(\vec{\omega}(T_{\rm F}))e^{-\frac{i}{\hbar}\int_0^{T_{\rm F}} E(\vec{\omega}(t'))dt'}$, in a short time $T_{\rm F}$.

In FFST[5], we assume that the wave function of the speed-controlled dynamics has the form

$$\phi_m^{\rm FF}(t) = \phi_m(\vec{\omega}(t))e^{if_m(t)}e^{-\frac{i}{\hbar}\int_0^t E(\vec{\omega}(t'))dt'}, \tag{S22}$$

and $\phi_m^{\rm FF}$ is a solution of the Schrödinger equation:

$$i\frac{d}{dt}\phi_m^{\rm FF}(t) = \sum_{l(\neq m)}^{N} g_{ml}\phi_l^{\rm FF}(t) + \omega_m^{\rm FF}(t)\phi_m^{\rm FF}(t). \tag{S23}$$

In the same manner as earlier, we can obtain

$$\frac{d\phi_m(\vec{\omega}(t))}{dt} = \sum_{l(\neq m)}^{N} g_{ml}\phi_l(\vec{\omega}(t))\sin[f_l(t) - f_m(t)], \tag{S24}$$

and

$$\omega_m^{\rm FF}(t) = \omega_m(t) + \sum_{l(\neq m)}^{N} g_{ml}\frac{\phi_l(\vec{\omega}(t))}{\phi_m(\vec{\omega}(t))}\{1 - \cos[f_l(t) - f_m(t)]\} - \frac{df_m(t)}{dt}, \tag{S25}$$

where we assumed that $\phi_m(\vec{\omega}(t))$ is real. Equation (S24) is used to calculate the additional phase, $f_m(t)$, while Eq. (S25) is used to calculate the control parameter, $\omega_m^{\rm FF}$. We set $f_1(t) = 0$ as we did in the main text.

As an example, we consider a three-level system with $g_{12} = g_{23} = g$, $g_{13} = 0$ and $g_{ml} = g_{lm}$. This system can also effectively describe the dynamics of three qubits coupled by strengths g_{ij}, when there is no interaction between qubits 1 and 3 and when the dynamics of the system is confined in the subspace spanned only by three states, $|100\rangle$, $|010\rangle$ and $|001\rangle$. (The Schrödinger equation (S20) can also describes the dynamics of Bose-Einstein condensates in an optical lattice[6–8].) The time dependence of the control parameters in the unmodified dynamics is given by $\omega_m(t) = m\tilde{\omega}(t)$, where

$$\tilde{\omega}(t) = \frac{\Delta\omega}{T_{\rm F}}\left(t - \frac{T_{\rm F}}{2\pi}\sin(2\pi t/T_{\rm F})\right) + \tilde{\omega}_0. \tag{S26}$$

We set $\Delta\omega = 3g$ and $\tilde{\omega}_0 = -1.5g$. The initial state is in the ground state. The time dependence of ω_m and the population, $|\phi_m|^2$, of each level for the ground state (target state) are presented in Fig. S4. Figure S5 shows the time dependence of the population, $|\Psi_m|^2$, of each level in the unmodified control with $\omega_m(t)$ for $T_{\rm F} = 6.0g^{-1}$ and $2.6g^{-1}$. It is seen that the populations deviate from the ones of the ground state. The fidelity of the unmodified control is 0.94 for $T_{\rm F} = 6.0g^{-1}$ and 0.66 for $T_{\rm F} = 2.6g^{-1}$, respectively.

We define $\beta_{\rm STA}^{\rm FF(i)}$ as

$$\beta_{\rm STA}^{\rm FF(1)}(t, f_2) = \frac{d\phi_1(\vec{\omega}(t))}{dt} - g\phi_2(\vec{\omega}(t))\sin[f_2], \tag{S27}$$

$$\beta_{\rm STA}^{\rm FF(2)}(t, f_2, \Delta f_{32}) = \frac{d\phi_2(\vec{\omega}(t))}{dt} + g\phi_1(\vec{\omega}(t))\sin[f_2] - g\phi_3(\vec{\omega}(t))\sin[\Delta f_{32}], \tag{S28}$$

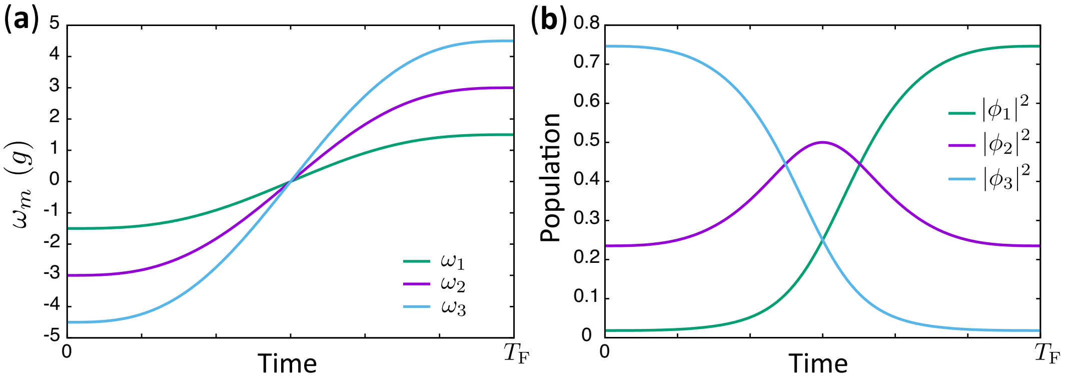

Figure S4. Time dependence of ω_m (a) and the population $|\phi_m|^2$ of each level for the ground state (target state) (b). The used parameters are $\Delta\omega = 3g$, $\tilde{\omega}_0 = -1.5g$.

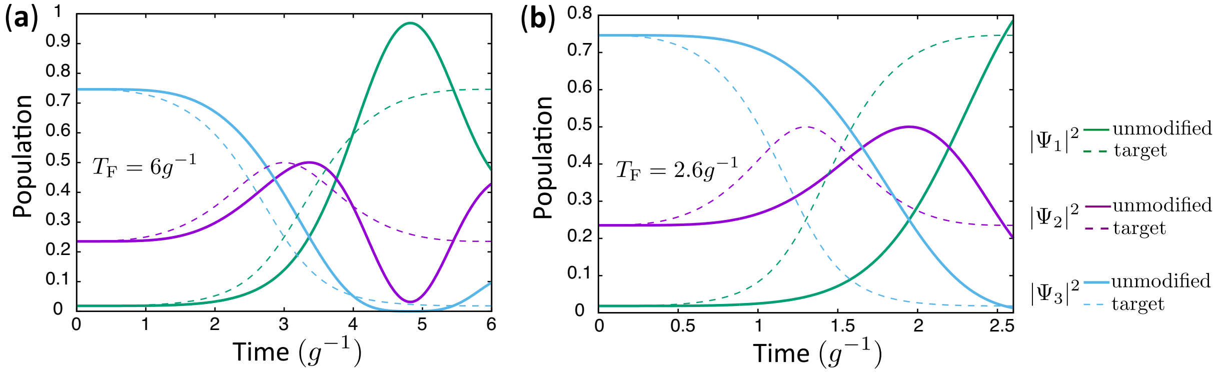

Figure S5. Time dependence of the population of each level in the unmodified control with $\omega_m(t)$ for $T_F = 6g^{-1}$ (a) and $T_F = 2.6g^{-1}$ (b). The dashed curves are for the populations of the ground state (target state). The used parameters are $\Delta\omega = 3g$, $\tilde{\omega}_0 = -1.5g$.

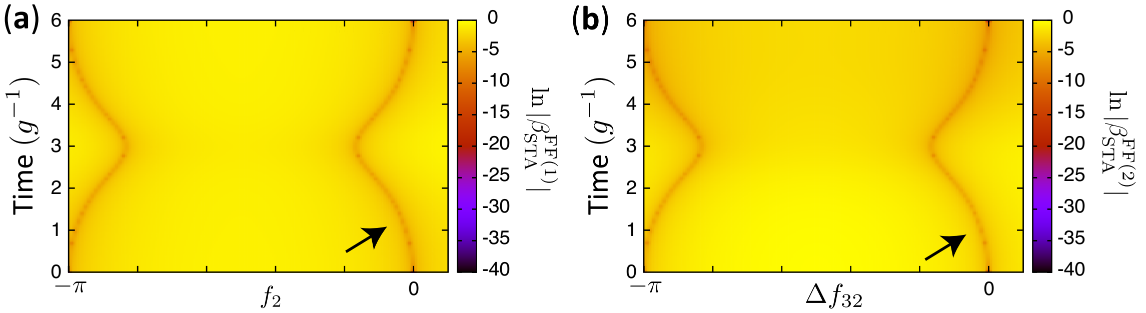

Figure S6. (a) $\ln|\beta_{\rm STA}^{\rm FF(1)}(t,f_2)|$ as a function of t and f_2. (b) $\ln|\beta_{\rm STA}^{\rm FF(2)}(t,f_2(t),\Delta f_{32})|$ as a function of t and Δf_{32}, where $f_2(t)$ was chosen to be a solution of $\beta_{\rm STA}^{\rm FF(1)}(t,f_2)=0$. The arrows indicate the relevant speed-controlled trajectory, which satisfies $f_2(0)=f_2(T_{\rm F})=\Delta f_{32}(0)=\Delta f_{32}(T_{\rm F})=0$. The used parameters are $\Delta\omega=3g$, $\tilde{\omega}_0=-1.5g$ and $T_{\rm F}=6g^{-1}$.

where Δf_{32} is a variable corresponding to f_3-f_2 of Eq. (S24). $f_2(t)$ and $\Delta f_{32}(t)$, which satisfy $\beta_{\rm STA}^{\rm FF(1)}=\beta_{\rm STA}^{\rm FF(2)}=0$, correspond to a viable speed-controlled trajectory, because Eq. (S24) holds for such $f_2(t)$ and $\Delta f_{32}(t)$. Figure S6(a) shows $\ln|\beta_{\rm STA}^{\rm FF(1)}(t,f_2)|$ as a function of t and f_2 for $T_{\rm F}=6g^{-1}$. Figure S6(b) shows $\ln|\beta_{\rm STA}^{\rm FF(2)}(t,f_2(t),\Delta f_{32})|$ as a function of t and Δf_{32}, where $f_2(t)$ was chosen to be a solution of $\beta_{\rm STA}^{\rm FF(1)}(t,f_2)=0$. It is seen that there is a set of $f_2(t)$ and $\Delta f_{32}(t)$ which satisfies $\beta_{\rm STA}^{\rm FF(1)}=\beta_{\rm STA}^{\rm FF(2)}=0$ and $f_2(0)=f_2(T_{\rm F})=\Delta f_{32}(0)=\Delta f_{32}(T_{\rm F})=0$. Therefore, there is a viable speed-controlled trajectory for $T_{\rm F}=6g^{-1}$.

In contrast to the case of $T_{\rm F}=6g^{-1}$, there is no viable speed-controlled trajectories for $T_{\rm F}=2.6g^{-1}$. In Figs. S7(a) and S7(b), $|\beta_{\rm STA}^{\rm FF(1)}(t,f_2)|$ is presented as a function of t and f_2 for $T_{\rm F}=2.6g^{-1}$. We introduce a virtual trajectory for f_2, defined by $f_2(t)=-\eta_1^{(2)}\exp[-\eta_2^{(2)}(t-T_{\rm F}/2)^2]$ with $\eta_1^{(2)}=1.35$ and $\eta_2^{(2)}=3.33g^2$, where $\eta_i^{(2)}$ was chosen so that $\int_0^{T_{\rm F}}|\beta_{\rm STA}^{\rm FF(1)}(t,f_2(t))|dt$ is minimized. Figures S7(c) and S7(d) show $|\beta_{\rm STA}^{\rm FF(2)}(t,f_2(t),\Delta f_{32})|$ as a function of t and Δf_{32}, where $f_2(t)$ was chosen to be the virtual trajectory of f_2. Now, we introduce a virtual trajectory for $\Delta f_{32}(t)$, defined by $\Delta f_{32}(t)=-\eta_1^{(32)}\exp[-\eta_2^{(32)}(t-T_{\rm F}/2)^2]$ with $\eta_1^{(32)}=1.29$ and $\eta_2^{(32)}=3.16g^2$, where $\eta_i^{(32)}$ was chosen so that $\int_0^{T_{\rm F}}|\beta_{\rm STA}^{\rm FF(2)}(t,f_2(t),\Delta f_{32}(t))|dt$ is minimized.

Control parameters $\omega_m^{\rm FF}$ in Eq. (S25) are obtained using the viable and virtual trajectories for $T_{\rm F}=6g^{-1}$ and $T_{\rm F}=2.6g^{-1}$, respectively. Figure S8 shows the time dependence of $\omega_m^{\rm FF}$. The modulation of the control parameters for $T_{\rm F}=2.6g^{-1}$ is rapid compared to that for $T_{\rm F}=6g^{-1}$ due to the rapidly changing f_i. The fidelity of the control with FFST for $T_{\rm F}=6g^{-1}$ and the control with ITT for $T_{\rm F}=2.6g^{-1}$ is unity and 0.998, respectively, while the fidelity of the unmodified control is 0.94 and 0.66 for $T_{\rm F}=6g^{-1}$ and $T_{\rm F}=2.6g^{-1}$, respectively, as mentioned earlier.

References

1. Koch, J., et al. Charge-insensitive qubit design derived from the cooper pair box. *Phys. Rev. A* **76,** 042319 (2007).
2. Didier, N., Sete, E. A., da Silva, M. P. & Rigetti, C. Analytical modeling of parametrically modulated transmon qubits. *Phys. Rev. A* **97,** 022330 (2018).
3. Hutchings, M. D., et al. Tunable superconducting qubits with flux- independent coherence, *Phys. Rev. Applied* **8,** 044003 (2017).
4. Blais, A., Grimsmo, A. L., Girvin, S. M. & Wallraff, A. Circuit quantum electrodynamics. *Rev. Mod. Phys.* **93,** 25005 (2021).
5. Masuda, S. & Rice, S. A. Rapid coherent control of population transfer in lattice systems. *Phys. Rev. A* **8,** 033621 (2014).
6. Trombettoni, A. & Smerzi, A. Variational dynamics of Bose-Einstein condensates in deep optical lattices, *J. Phys. B: At. Mol. Opt. Phys.* **34,** 4711–4720 (2001).
7. Trombettoni, A. & Smerzi, A. Discrete Solitons and Breathers with Dilute Bose-Einstein Condensates, *Phys. Rev. Lett.* **86,** 2353 (2001).
8. Cataliotti, F. S., Burger, S., Fort, C., Maddaloni, P., Minardi, F., Trombettoni, A., Smerzi, A., & Inguscio, M. Josephson Junction Arrays with Bose-Einstein Condensates, *Science* **293,** 843 (2001).

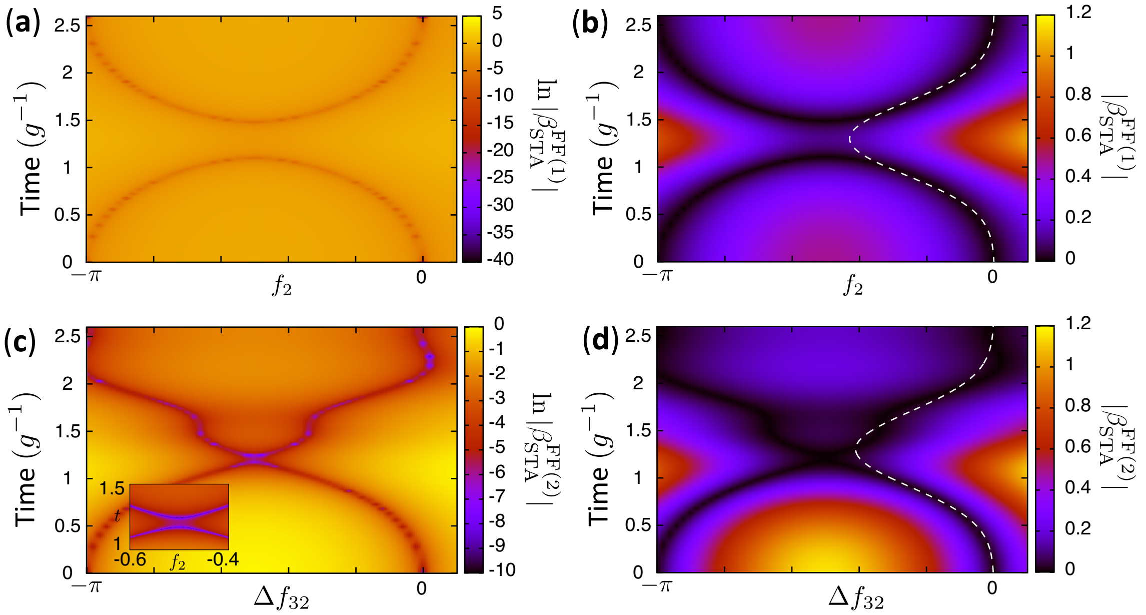

Figure S7. (a,b), $\ln|\beta_{\mathrm{STA}}^{\mathrm{FF}(1)}(t, f_2(t))|$ and $|\beta_{\mathrm{STA}}^{\mathrm{FF}(1)}(t, f_2(t))|$ as a function of t and f_2. (c,d), $\ln|\beta_{\mathrm{STA}}^{\mathrm{FF}(2)}(t, f_2(t), \Delta f_{32})|$ and $|\beta_{\mathrm{STA}}^{\mathrm{FF}(2)}(t, f_2(t), \Delta f_{32})|$ as a function of t and Δf_{32}, where $f_2(t)$ was chosen to be the virtual trajectory of f_2. The inset of panel (c) is a closeup, which shows a narrow gap between trajectories. The used parameters are $\Delta\omega = 3g$, $\tilde{\omega}_0 = -1.5g$ and $T_{\mathrm{F}} = 2.6g^{-1}$.

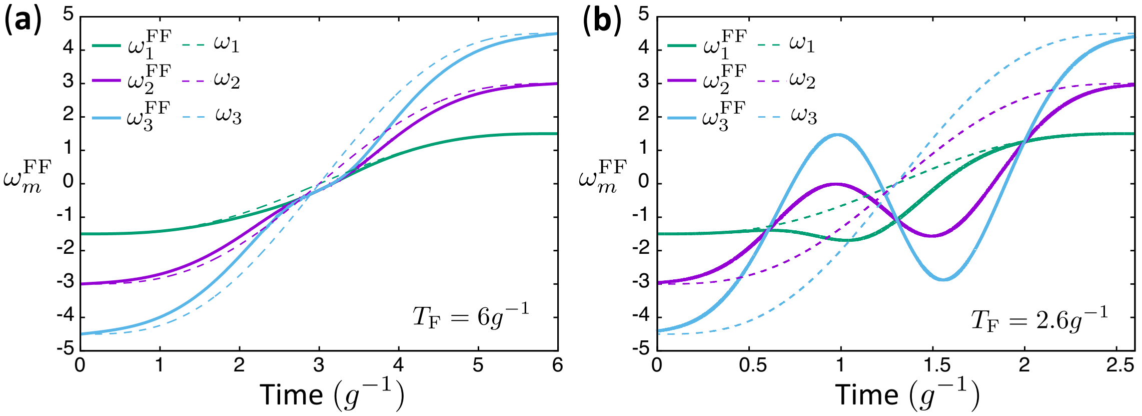

Figure S8. ω_m^{FF} as a function of t for $T_{\mathrm{F}} = 6g^{-1}$ (a) and $T_{\mathrm{F}} = 2.6g^{-1}$ (b). The used parameters are $\Delta\omega = 3g$ and $\tilde{\omega}_0 = -1.5g$.
